# Long-term monitoring of opioid, sedative and anti-inflammatory drugs in horse hair using a selective and sensitive LC-MS/MS procedure

**DOI:** 10.1186/s12917-016-0709-5

**Published:** 2016-06-01

**Authors:** Milena M. Madry, Barbara S. Spycher, Jacqueline Kupper, Anton Fuerst, Markus R. Baumgartner, Thomas Kraemer, Hanspeter Naegeli

**Affiliations:** Zurich Institute of Forensic Medicine, Center for Forensic Hair Analytics, University of Zurich, Zurich, Switzerland; Zurich Institute of Forensic Medicine, Center for Forensic Pharmacology and Toxicology, University of Zurich, Zurich, Switzerland; Institute of Veterinary Pharmacology and Toxicology, University of Zurich, Zurich, Switzerland; Clinic of Veterinary Surgery, Department of Large Animal Surgery, University of Zurich, Zurich, Switzerland

**Keywords:** Doping, LC-MS/MS, Pre-purchase examination, Horse hair

## Abstract

**Background:**

Compared to blood or urine, drugs can be detected for much longer periods in the long hair of horses. The aim of this study was to establish and validate a highly sensitive liquid chromatography tandem mass spectrometry (LC-MS/MS) method for the detection and quantification of frequently prescribed opioids, sedatives and non-steroidal anti-inflammatory agents in the mane and tail hair of horses. Based on an average growth rate of about 2 cm per month, times of administration reported by horse owners or veterinary physicians were related to drug localizations in hair. Hair samples were collected from ten horses that received drug treatments and analyzed in segments of 2, 4 or 6 cm in length. Hair segments were decontaminated, cut into fragments and methanol-extracted under sonication. The extracts were analyzed by LC-MS/MS for 13 commonly used drugs using the validated procedure. Deuterated analogs were included as internal standards.

**Results:**

Analytes were detected in hair samples with a length of up to 70 cm. Fourteen out of 16 hair samples were positive for at least one of the tested drugs. Segmentation allowed for time-resolved monitoring of periods of 1 to 3 months of drug administration. Concentrations in dark hair reached a maximum of 4.0 pg/mg for butorphanol, 6.0 pg/mg for tramadol, 1.4 pg/mg for morphine, 1.8 pg/mg for detomidine, 1.2 pg/mg for acepromazine, 39 pg/mg for flunixin, 5.0 pg/mg for firocoxib, and 3’600 pg/mg for phenylbutazone. Only trace amounts of meloxicam were detected. Drug detection correlated well with the reported period of medical treatment. No analytes were detected in the light-colored mane and tail hair samples from one horse despite preceding administrations of acepromazine and phenylbutazone.

**Conclusion:**

This study describes a sensitive and selective technique suitable for the validated detection and quantification of frequently prescribed veterinary drugs in horse hair. The segmental method can be applied for time-resolved long-term retrospective drug monitoring, for example in prepurchase examinations of horses as drug detection in hair can prove preceding medical treatments.

## Background

Horse hair analysis has gained increasing interest for the monitoring of veterinary drugs, hormones, nutrients, trace elements and contaminants [1–7]. In contrast to blood and urine, hair provides a long-term historical record of drug exposure that may be useful for applications in sports anti-doping control programs, pre-purchase examination of horses and examinations for insurance purposes [2]. Other advantages of hair analyses include the non-invasive probing as well as drug-stability in hair and easy shipping of samples [8]. The growth of temporary hair of the coat changes with seasons whereas the permanent mane and tail hair grows continuously with relatively constant rates reported in the range from 1.7 to 2.5 cm per month [1, 2, 9, 10]. Therefore, samples of long hair can be segmented to narrow down the corresponding times of drug exposure [2, 11].

So far, detection in horse hair has been demonstrated for morphine [1], clenbuterol [3], anabolic steroids [4, 7, 9], antimicrobial agents [12], diazepam [13], cortisol [14], arsenic [6] and selenium [15]. The aim of this study was twofold. The first goal was to extend the use of mane and tail hair as a matrix for the monitoring of sedative, analgesic and anti-inflammatory drugs that are frequently prescribed to horses. The second goal was to compare closely the analytical findings in hair segments with the corresponding times of drug administration. To that end, a highly sensitive liquid chromatography-tandem mass spectrometry (LC-MS/MS) method was established, validated and applied to mane and tail hair samples of 10 horses with a known history of pharmacologic treatments.

## Methods

### Study design and sample collection

The study included ten horses with documented administration of at least one of the investigated drugs before sampling. All horse owners provided informed consent to participate in the study. Sixteen mane and nine tail hair samples were collected by horse owners or veterinarians. One tail hair sample was collected post-mortem. The sampling method was adapted from guidelines of the Society of Hair Testing (SoHT) [11]. A hair lock was fixed with the hair strands equally aligned using a string and cut precisely close to the skin. The lock was wrapped in aluminum foil with the proximal ends aligned for clear identification. Samples were stored at room temperature under dry conditions and in the dark. Individual data on drug administration, hair type and color of each horse are reported in Table 1.

**Table 1:** Drug treatment of the horses (relative to time of sample collection) and characteristics of hair samples

Horse	Reported drug (s)	Time before sampling	Hair samples (length, color)
P01	Acepromazine	2 months	Mane (16 cm, light) Tail (28 cm, light)
	Phenylbutazone	3 and 9 months	
P02	Butorphanol	22 months	Tail (70 cm, dark)
	Detomidine	With euthanasia	
	Flunixin	1 day and 22 months	
P03	Flunixin	8 months	Mane (16 cm, dark) Tail (28 cm, dark)
	Ketoprofen	8 months	
P04	Flunixin	9 months	Mane (16 cm, dark) Tail (26 cm, dark)
	Ketoprofen	9 months	
	Phenylbutazone	10 months	
P05	Flunixin	7 months	Mane (16 cm, dark) Tail (28 cm, dark)
P06	Acepromazine	15 months	Tail (68 cm, dark)
	Butorphanol	17 months	
	Phenylbutazone	8 months	
P07	Flunixin	2 and 4 months	Mane (24 cm, dark) Tail (26 cm, dark)
	Phenylbutazone	2 and 4 months	
P08	Butorphanol	6 months	Mane (20 cm, dark)
	Detomidine	6 months	
	Phenylbutazone	10 months	
	Tramadol	6 months	
P09	Flunixin	1 week	Mane (24 cm, white) Tail (24 cm, dark)
	Phenylbutazone	1 week	
P10	Butorphanol	8 months	Tail (44 cm, dark)
	Firocoxib	10 months	
	Meloxicam	10 months	

### Analytical standards and chemicals

Morphine, morphine-d_3_, tramadol, ^13^C-tramadol-d_3_, buprenorphine-d_4_, chlorpromazine-d_3_, and phenylbutazone were obtained as solutions from Sigma-Aldrich Chemie GmbH (Buchs, Switzerland). Butorphanol and buprenorphine were obtained as solutions from ReseaChem GmbH (Burgdorf, Switzerland) and Lipomed AG (Arlesheim, Switzerland), respectively. Ketoprofen-d_3_ and phenylbutazone-(diphenyl^13^C_12_) were purchased as powders Sigma-Aldrich Chemie GmbH (Buchs, Switzerland). Detomidine, acepromazine, meloxicam, ketoprofen, chlorpromazine, flunixin and fluphenazine were analytical standards available as powders in the authors’ laboratory. All solvents and other chemicals were purchased from Sigma-Aldrich Chemie GmbH (Buchs, Switzerland). All standards were dissolved in methanol, except for meloxicam that was dissolved in dimethylformamide, to yield stocks of 1 mg/ml. Firocoxib was obtained as an injectable for veterinary use. Chromatography-grade water used for LC-MS/MS analysis was processed by a PURELAB Option-Q system by ELGA LabWater (Labtec Services AG, Villmergen, Switzerland).

### Preparation of working solutions

Spiking solutions for calibrators and quality control (QC) samples were prepared in methanol to yield concentrations comparable to those found in hair. An internal standard (IS) solution with the following concentrations was prepared in methanol: morphine-d_3_ (15 ng/ml), ^13^C-tramadol-d_3_ (8.0 ng/ml), buprenorphine-d_4_ (20 ng/ml), ketoprofen-d_3_ (10 ng/ml), chlorpromazine-d_3_ (10 ng/ml) and phenylbutazone-(diphenyl^13^C_12_) (30 ng/ml).

### Hair sample preparation

Mane and tail hair samples were cut into 2- or 4-cm segments; one very long hair sample of 70 cm was analyzed in 6-cm segments. For a thorough decontamination according to the laboratory standard procedure for forensic hair analysis, 10 mg of hair were washed once with 5 mL deionized water and twice with 5 mL acetone for 3 min each. After drying at room temperature, hair segments were chopped in snippets with scissors. For extraction, 100 μl IS solution and 1900 μl methanol were added and samples were sonicated for 4 h at 50 °C. After centrifugation for 7.5 min at 10,000 g, 1500 μl of the clear solution was transferred into a vial for evaporation under a stream of nitrogen at 40 °C. For injection into the LC-MS/MS system, the residue was reconstituted in 60 μl 5 mM ammonium formate^a^ (pH 3) with 10 % (*v/v*) methanol.

### LC-MS/MS parameters

Analytes were separated on a C18 column (Phenomenex Kinetex 2.6 μm C18 column, 50 × 2.1 mm, Brechbühler AG, Schlieren, Switzerland) using a high-performance LC system (Ultimate 3000 HPLC system, Thermo Fisher Scientific AG, Reinach, Switzerland) and detected by a linear ion trap triple quadrupole mass spectrometer (Applied Biosystems 5500 Q Trap with Analyst software, Sciex, Darmstadt, Germany). The mobile phase consisted of 5 mM ammonium formate buffer (pH 3; eluent A) and methanol containing 5 mM ammonium formate (eluent B). The following gradient was used: 10 to 50 % B from 0 to 3 min, 50 % B from 3 to 10 min, 50 to 90 % B from 10 to 10.5 min and 90 % B from 10.5 to 11.5 min. The flow rate was 0.35 ml/min. The column temperature was 35 °C and injection volumes were 10 μl. The MS instrument was operated in positive electrospray ionization mode. Three specific multiple reaction monitoring (MRM) transitions per substance were analyzed, but for detomidine only two transitions could be recorded (Table 2). Quantification was achieved using the most abundant transition of each precursor to the respective product ion. Criteria for positive identification were the matching retention time, three specific MRM transitions (one served as quantifier and two served as qualifier), and the matching relative ion intensities of MRM transitions (qualifier-to-quantifier) which were determined from calibrators (*n* = 10 each) (Table 2). The tolerance level for percent deviations of relative intensities of MRM transitions was ±30 % [16]. The absence of interfering peaks was verified for all analytes in blank samples and after addition of the IS solution.

**Table 2:** LC-MS/MS parameters

Analyte/internal standard	Precursor ion (m/z)	Product ion (m/z)	Relative ion intensities (mean) ± rSD (%)^a^	RT (min)	DP (V)	EP (V)	CE (V)	CxP (V)
Morphine	286.1	**152.1**	0.7 ± 16	0.6	156	10	81	14
		128.1		0.6	156	10	79	16
		165.1		0.6	156	10	57	16
Tramadol	264.05	**58.0**	0.32 ± 6.2	3.4	46	10	17	10
		58.5		3.4	46	10	33	8
		42.0		3.4	46	10	115	20
Detomidine	187.09	**81.1**	0.23 ± 7.7	3.9	101	10	25	8
		54.1		3.9	101	10	55	8
Butorphanol	328.17	**310.2**	0.15 ± 3.6	4.2	101	10	33	22
		157.0		4.2	101	10	59	12
		131.1		4.2	101	10	65	12
Buprenorphine	468.24	**55.1**	0.05 ± 12	5.1	1	10	101	6
		41.1		5.1	1	10	129	4
		115.1		5.1	1	10	129	6
Acepromazine	327.04	**86.0**	0.99 ± 5.6	5.5	81	10	25	10
		58.0		5.5	81	10	65	8
		222.1		5.5	81	10	53	16
Firocoxib	337.02	**283.1**	0.81 ± 7.1	5.6	101	10	13	16
		130.1		5.6	101	10	41	12
		237.0		5.6	101	10	23	20
Meloxicam	351.96	**115.0**	0.37 ± 7.0	6.3	126	10	25	10
		141.0		6.3	126	10	27	12
		73.0		6.3	126	10	75	12
Chlorpromazine	319.26	**58.1**	0.89 ± 4.2	6.7	86	10	67	12
		86.1		6.7	86	10	25	8
		214.0		6.7	86	10	57	20
Ketoprofen	255.08	**77.0**	0.93 ± 6.7	6.7	131	10	61	12
		104.9		6.7	131	10	33	12
		51.1		6.7	131	10	99	8
Flunixin	297.03	**278.9**	0.38 ± 3.1	7.3	111	10	33	24
		264.0		7.3	111	10	47	22
		236.1		7.3	111	10	57	16
Fluphenazine	438.22	**171.0**	0.71 ± 2.8	9.3	56	10	37	20
		143.1		9.3	56	10	37	18
		70.0		9.3	56	10	67	10
Phenylbutazone	309.15	**77.0**	0.51 ± 9.6	9.5	216	10	75	12
		160.1		9.5	216	10	29	14
		120.0		9.5	216	10	27	10
Morphine-d_3_	289.02	**201.0**		0.6	156	10	35	16
		152.1		0.6	156	10	75	18
^13^C-Tramadol-d_3_	268.07	**58.0**		3.4	46	10	49	14
		42.1		3.4	46	10	113	20
Buprenorphine-d_4_	472.26	**59.0**		5.1	1	10	107	6
		400.1		5.1	1	10	53	2
Chlorpromazine-d_3_	322.04	**89.1**		6.7	51	10	25	10
		61.1		6.7	51	10	59	8
Ketoprofen-d_3_	258.09	**212.1**		6.7	121	10	19	14
		105.0		6.7	121	10	31	10
^13^C_12_-Phenylbutazone	321.11	**166.2**		9.5	76	10	29	14
		55.1		9.5	76	10	129	8

MRM (multiple reaction monitoring mode) transitions with precursor and product ions, relative ion intensities of MRM transitions (qualifier-to-quantifier); *RT* retention time, *DP* declustering potential, *EP* entrance potential, *CE* collision energy, *CxP* collision cell exit potential. MRM transitions used as quantifiers are given in bold

^a^determined from calibrators (*n* = 10, per analyte)

### Method validation

The method was validated for selectivity, limit of detection (LOD), lower limit of quantification (LLOQ), linearity of calibration, accuracy, intra-day and inter-day precision, and matrix effects based on the Guidelines for Quality Control in Forensic-Toxicological Analyses [17, 18]. All validation parameters were assessed with drug-free hair samples (blank hair) which were obtained from horses that had never received medications. Drug-free hair was spiked with 100 μl IS solution and 100 μl calibrator or QC solution. The calibration line was calculated using a weighted [1/x, x = f (concentration)] linear regression model. Calibration curves with seven calibrators were prepared for eight days. LOD was defined as the lowest concentration with a peak height showing a signal-to-noise ratio of at least 3:1 in the chromatogram, (Table 3). The LLOQ showing a signal-to-noise of at least 10:1 was chosen as the lowest calibrator concentration; LLOQ levels ranged from 0.1 to 5 pg/mg, except for phenylbutazone for which an LLOQ of 25 pg/mg was determined. Accuracy and precision were determined via daily calibration curves over eight days by analyzing duplicate QC samples at low, medium, and high concentrations (Table 3). The accepted intervals for precision were ± 15 % (±20 % at LLOQ) for bias and standard deviation of the nominal concentration. Processed sample stability in the auto-sampler at room temperature was tested by pooling extracts prepared at these low and high concentrations. Six aliquots were injected in the LC-MS/MS system every 2 h over 18 h. Further stability studies were not included as samples were tested immediately after their preparation. Matrix effects were studied with hair samples of different sources as previously proposed [17, 19]: the peak area of analytes in blank hair samples of different horses (*n* = 5) spiked after the extraction was divided by the mean peak area of analytes in neat standard solutions (*n* = 5) at the same concentration level and multiplied by 100. Values below or above 100 indicate ion suppression or ion enhancement resulting in diminished or increased signal intensities.

**Table 3:** Analyte concentrations in method validation: for limit of detection (LOD), lower limit of quantification (LLOQ), calibration range and quality control (QC) samples

Analyte	LOD (pg/mg)	LLOQ (pg/mg)	Cal. range (pg/mg)	QC low (pg/mg)	QC medium (pg/mg)	QC high (pg/mg)
Morphine	0.1	0.6	0.6–840	0.585	270	555
Tramadol	0.1	0.5	0.5–100	0.48	30	68
Detomidine	0.1	0.6	0.6–36	0.585	11	24
Butorphanol	0.1	0.5	0.5–50	0.48	15	33
Buprenorphine	1	2	2–800	1.95	225	540
Acepromazine	0.05	0.1	0.1–150	0.098	45	101
Firocoxib	0.5	1	1–740	0.975	225	495
Meloxicam	0.5	2	2–280	1.95	90	188
Ketoprofen	1	5	5–420	4.8	135	278
Chlorpromazine	0.5	1	1–280	0.975	90	189
Flunixin	0.05	0.1	0.1–66	0.098	223	44
Fluphenazine	0.5	1	1–600	0.975	180	405
Phenylbutazone	10	25	25–7000	24	2250	4650

## Results

### Method validation

**Fig. 1:**
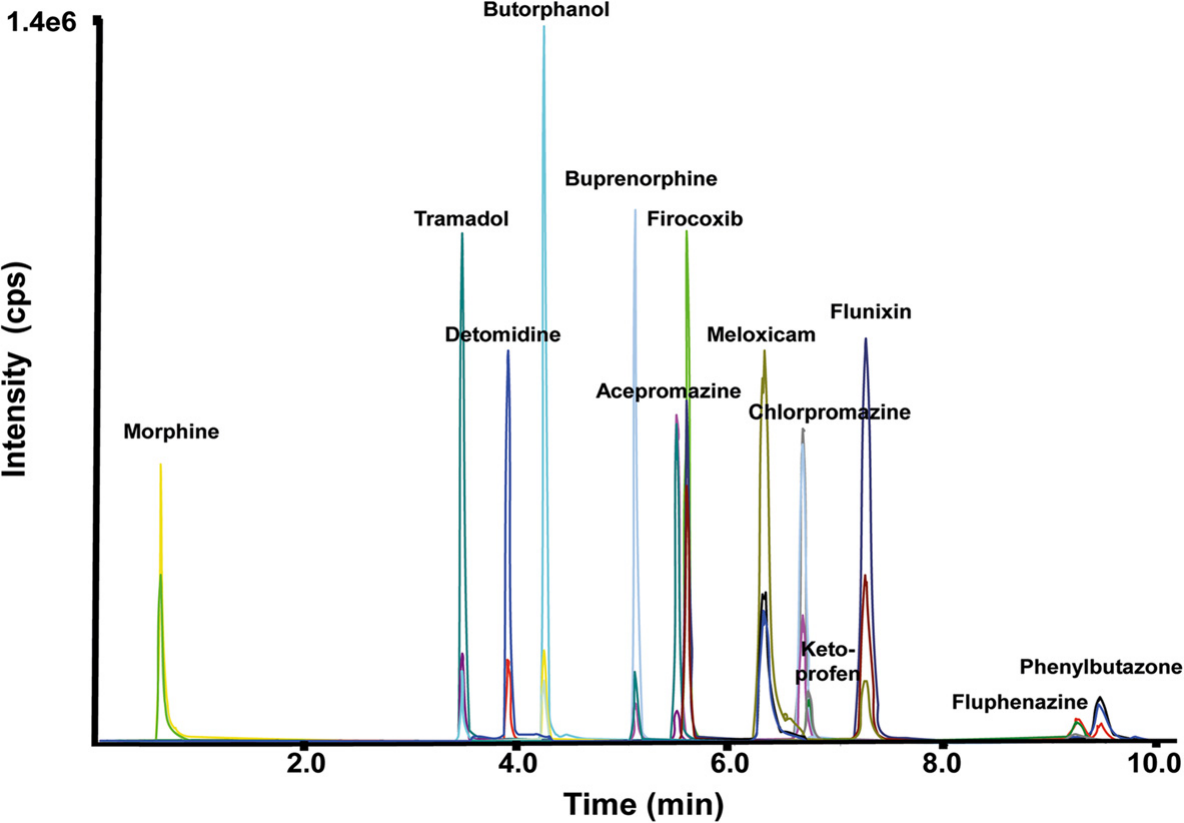
LC-MS/MS chromatogram of a spiked sample with the respective MRM transitions for 13 investigated drugs

An LC-MS/MS method was developed for the detection and quantification of 13 sedative, analgesic and anti-inflammatory drugs commonly used in horses. A representative chromatogram including all analytes is given in Fig. 1. The method, validated according to the Guidelines for Quality Control in Forensic-Toxicological Analyses [17, 18], was selective and very sensitive for all analytes. The detection of buprenorphine, butorphanol, detomidine, firocoxib, flunixin, ketoprofen, meloxicam, morphine and phenylbutazone fulfilled all validation criteria (Table 4). For chlorpromazine and tramadol, matrix effects exceeded the limits of ± 25 % standard deviation, but this was compensated by the inclusion of deuterated standards. The detection of acepromazine and fluphenazine displayed deviations in accuracy as well as precision, and variable matrix effects were observed. However, inclusion of labeled analogs of the two compounds allowed for comparisons between samples from the same horse. For all other drugs, the method was successfully validated and proven to be suitable for the comparison of hair samples from different horses.

**Table 4:** Validation parameters: accuracy, intra-day and inter-day precision, matrix effects. N/A, not determined

Analyte	QC sample	Accuracy, bias (%)	Intra-day precision, SD (%)	Inter-day precision, SD (%)	ME, mean ± SD (%)
Morphine	Low	0.6	6.2	8.8	56 ± 6.9
	Medium	3.2	3.5	3.8	N/A
	High	−0.1	3.4	3.4	53 ± 9.8
Tramadol	Low	2.4	4.3	5.2	107 ± 21
	Med	0.4	4.5	4.5	N/A
	High	4.9	1.8	2.3	183 ± 90
Detomidine	Low	3.9	7.6	7.7	88 ± 24
	Medium	5.9	7.7	9.1	N/A
	High	2.9	4.5	5.9	79 ± 12
Butorphanol	Low	−0.4	10.2	15.2	87 ± 12
	Medium	1.9	4.5	4.5	N/A
	High	−1.8	4.0	6.6	84 ± 10
Buprenorphine	Low	−9.3	4.5	8.0	87 ± 23
	Medium	−1.2	4.5	4.5	N/A
	High	−0.6	2.0	2.0	88 ± 7.7
Acepromazine	Low	87	28	53	97 ± 31
	Medium	8.3	8.2	11	N/A
	High	−0.7	11	11	117 ± 14
Firocoxib	Low	4.7	3.0	6.7	82 ± 11
	Medium	2.6	1.7	2.0	N/A
	High	2.8	4.0	4.0	83 ± 8.7
Meloxicam	Low	4.1	2.9	5.7	78 ± 12
	Medium	4.2	3.1	5.2	N/A
	High	−4.1	3.1	3.5	70 ± 12
Chlorpromazine	Low	−3.5	3.2	6.3	77 ± 42
	Medium	6.2	2.9	2.9	N/A
	High	0.9	2.9	3.2	90 ± 41
Ketoprofen	Low	6.1	5.6	7.0	78 ± 13
	Medium	−1.9	1.8	2.1	N/A
	High	5.3	5.5	5.5	86 ± 12
Flunixin	Low	5.8	5.8	7.3	89 ± 16
	Medium	0.4	2.2	2.8	N/A
	High	3.5	3.2	3.2	75 ± 7.0
Fluphenazine	Low	7.3	5.6	14.8	92 ± 125
	Medium	12.2	20	21	N/A
	High	18.4	18.7	19.3	81 ± 51
Phenylbutazone	Low	6.1	9.8	10.7	75 ± 14
	Medium	1.2	4.4	6.1	N/A
	High	2.3	4.6	6.2	60 ± 6.7

### Detection of opioids

**Fig. 2:**
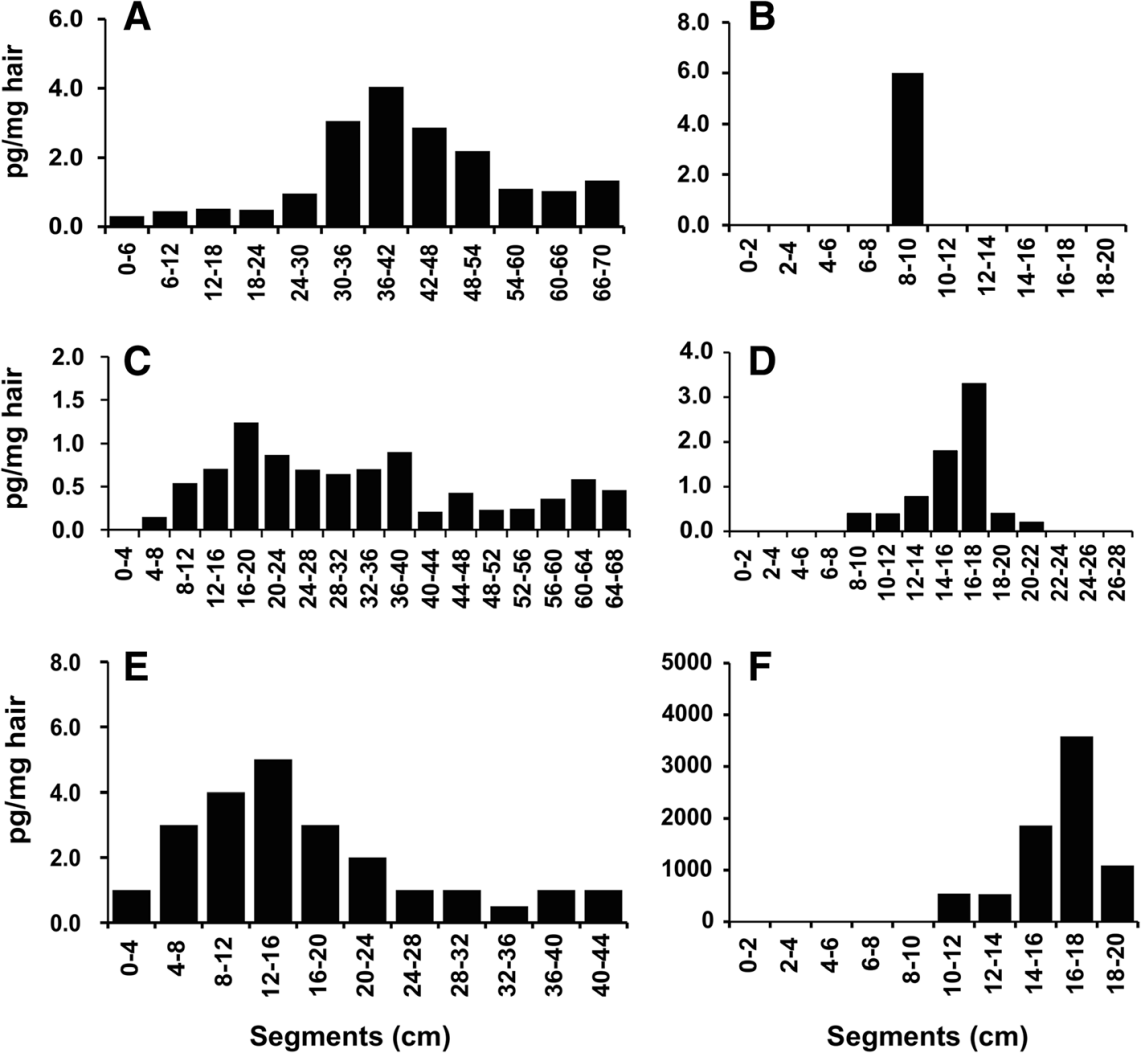
Drug localization patterns in the long hair of horses with documented treatments. **a** Distribution of butorphanol in tail hair 22 months after treatment. **b** Distinctive peak of tramadol in mane hair 6 months after treatment. **c** Distribution of acepromazine in tail hair 15–16 months after treatment. **d** Distribution of flunixin in tail hair 8 months after treatment. **e** Distribution of firocoxib in tail hair 10 months after treatment. **f** Distribution of phenylbutazone in mane hair 10 months after treatment

Butorphanol was detected in the hair of four out of five horses having received the drug. The concentrations ranged from below LLOQ to 4.0 pg/mg. In horse P02, repeated administrations of butorphanol were reported 22 months before sample collection and the highest concentration (4.0 pg/mg) was detected in a tail hair segment corresponding to a distance from the skin of 36 to 42 cm (Fig. 2a). In horse P08, a single application of butorphanol 6 months before sampling resulted in drug concentrations in tail hair of 0.9–2.3 pg/mg spread across a distance of 6–16 cm from the skin. In many cases, more drugs were found in hair than reported to have been administered. For example, butorphanol was detected in multiple 2-cm segments of the mane and tail hair of horse P07 (peak concentration of 3.6 pg/mg at a distance of 18–20 cm from the skin) and P10 (peak of 0.6 pg/mg at 22–24 cm from the skin), although administration of this drug was not reported. However, the presence of butorphanol was not demonstrated in the dark tail hair of horse P06 treated once 17 months before sample collection.

A single dose of tramadol was administered to horse P08 around 6 months before sample collection. This treatment resulted in a distinctive analyte peak (6 pg/mg) in a unique 2-cm segment of tail hair located 8–10 cm from the skin (Fig. 2b). A distinctive peak of the same drug (1.1 pg/mg) was also found in the tail hair of horse P02 but without any documentation of previous tramadol use. Morphine was present in the tail hair sample of horse P02 at concentrations of up to 1.4 pg/mg, although medication with this analgesic drug had not been recorded. There was no report of buprenorphine administration and, accordingly, all samples were negative for this substance.

### Detection of sedatives

A single detomidine application was given to horse P02 on the day of euthanasia. This resulted in detection of traces of the drug around or below the LLOQ in all tested mane and tail hair segments, indicating an external contamination by sweat or other biological fluids. Detomidine was also detected in the mane hair of horse P07, with maximum levels (1.8 pg/mg) at a distance of 4–6 cm from the skin, although administration of this drug was not reported. On the other hand, a single dose of detomidine 6 months before sampling could not be detected in the dark mane hair of horse P08. Acepromazine, given to horse P06 around 15–16 months before sampling, was retrieved at concentrations of 0.5–1.2 pg/mg in tail hair segments corresponding to a distance of 8–40 cm from the skin (Fig. 2c). There was no report of chlorpromazine and fluphenazine administration and none of the samples contained these drugs.

### Detection of non-steroidal anti-inflammatory agents

All hair samples of the four horses with reported flunixin administration were positive for this common anti-inflammatory agent, the highest detected concentration being 39 pg/mg. A sharp peak of flunixin deposition was observed in the tail hair of horse P03. In this case, drug administrations occurring 8 months before sampling yielded a distinctive accumulation at a distance of 16–18 cm from the skin (Fig. 2d). It should be noted that flunixin concentrations were higher in tail (up to 3.3 pg/mg) than in mane hair segments (below LLOQ). This trend was confirmed by comparing flunixin residues in the hair of horses P07 and P09 (data not shown). Firocoxib was detected in the tail hair of horse P10 at concentrations up to 5.0 pg/mg and at a distance of 12–16 cm from the (Fig. 2e). This residue is due to firocoxib treatments given 10 months before sampling.

Phenylbutazone administration was reported in six horses, but the substance was detected in the hair of only two of these animals. This is a consequence of the relatively high LOD (10 pg/mg) linked to poor ionization of phenylbutazone in the MS system. To our knowledge, incorporation of phenylbutazone into hair has not been described before. However, we were able to detect this drug along the entire tail hair of horse P06 with the highest concentration of 1300 pg/mg at a distance of 52–56 cm from the skin. This localization corresponds to a non-recorded treatment that may have been carried out more than 2 years before sampling. Phenylbutazone was also detected in the mane hair of horse P08 (Fig. 2f). In this case, the observed localization pattern with a peak concentration of 3600 pg/mg in the 16–18-cm segment is explained with known phenylbutazone administrations 10 months before sampling. Finally, meloxicam was detected in the tail hair of horse P02 at levels close to the LLOQ (data not shown). Ketoprofen had been administered in two cases but without leaving any detectable residues in hair.

### Influence of pigmentation

**Fig. 3:**
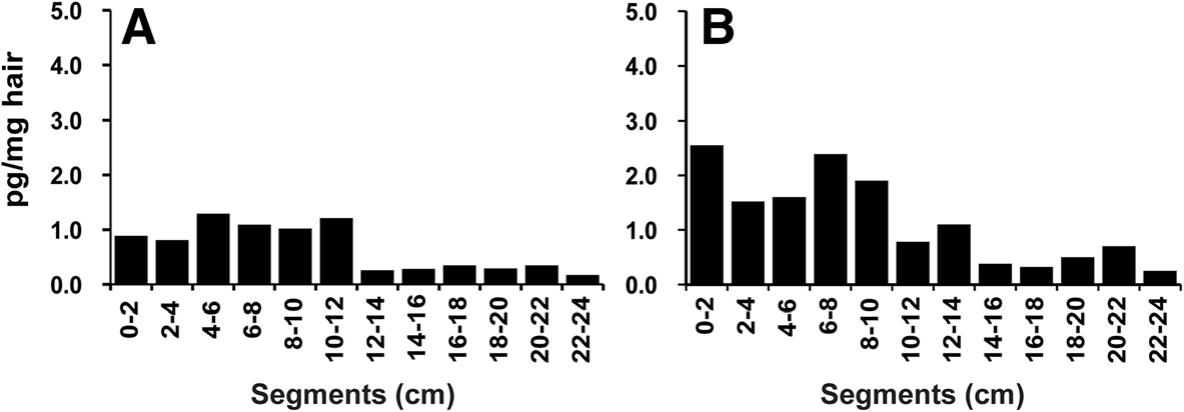
Pattern of flunixin in hair of the same horse treated 1 week before sample collection. **a** Flunixin in white mane hair. **b** Flunixin in dark tail hair. These findings reflect sweat- and sebum-mediated flunixin incorporation

The analyzed hair samples differed in color. The mane and tail hair of horse P01 as well as the mane hair of horse P09 were light and white, respectively, due to their low melanin content. In view of the melanin-dependent incorporation of basic drugs into hair (see Discussion), it is not surprising to find that the light hair of horse P01 was negative for all tested analytes although acepromazine and phenylbutazone administrations had been documented. Notably, the white mane hair of horse P09 (Fig. 3a) displayed only moderately lower concentrations of flunixin than the corresponding black tail hair of the same horse (Fig. 3b). However, all examined hair segments contained flunixin despite the fact that this drug was applied only 1 week before sample collection. Flunixin incorporated via the hair follicle could not yet have grown out as the follicle is located a few millimeters below the epidermis. Thus, the most likely explanation for the presence of flunixin in mane and tail hair of this horse is an incorporation via sweat and sebum [20].

### Correlation between time of treatment and hair segment

**Fig. 4:**
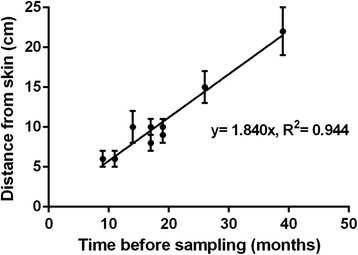
Drug localization in horse hair as a function of the time of documented drug administrations. This graph shows the median distance from skin and range of segments with clearly increased drug incorporation. The slop of the resulting linear relationship is consistent with an average growth rate of 1.84 cm per month

Frequently, the analysis of mane or tail hair segments revealed a clear peak of drug incorporation (see for example Fig. 2b). In case of a broader distribution, as for example in Fig. 2c, the median distance from the skin was taken as the measure for drug localization. Subsequently, the assignments of drug localization in hair were compared to the reported times of treatment (Fig. 4). This relationship demonstrated that the time of drug administration can be calculated by assuming an average hair growth rate of 1.84 cm per month.

## Discussion

The incorporation of a systemically administered drug into hair is dependent on the melanin content and the physicochemical properties of each substance [8, 21–23]. Basic drugs can reach 10-fold higher concentrations in pigmented than in non-pigmented hair [8, 21–23]. In contrast, no differences in hair incorporation were observed for acidic compounds and, as a consequence, hair concentrations of ketoprofen (pk_a_ = 4.45), meloxicam (pk_a_ = 4.05) or phenylbutazone (pk_a_ = 4.50) are low independently of hair color [8].

Several further considerations are necessary for the correct interpretation of hair findings. First, shifting of single hair against each other during sampling should be avoided to obtain the highest time resolution. Second, external contamination has to be taken into account and decontamination of hair samples is crucial. Third, one must consider that a substance may not only be found in the hair segment corresponding to the time of intake but also in adjacent segments. This may especially be the case when hair is cut into short segments of 1 to 2 cm length [8]. Fourth, the detection of substance in hair segments grown before drug administration can be explained by deposition mediated by sweat or sebum. Finally, it takes time for a hair sample to become negative after drug administration has been ceased due to the hair follicle proportion in the telogenic phase. In addition, hair follicle activity is compromised by disease or stressful events like anesthesia [24]. Decomposition of incorporated drug due to exposure to UV light or hair damage with drug leakage may account for negative findings [2].

## Conclusion

In conclusion, the present fully validated LC-MS/MS method offers a valuable tool for the retrospective tracking of drugs as their detection in a hair segment proves previous medical treatments dating back up to 22 months before sampling. It was demonstrated that, particularly but not exclusively, drugs with neutral or basic properties can be monitored in dark long hair of horses. Further, our study demonstrates that the time of drug administration can be estimated based on an average growth rate of 1.84 cm per month. Further investigations should include the analysis of metabolites. Moreover, it should be determined to what extent light hair displaying a low melanin content could also be used for drug monitoring.

## Abbreviations

LC-MS/MS, liquid chromatography tandem mass spectrometry; SoHT, society of hair testing; QC, quality control; MRM, multiple reaction monitoring; LOD, limit of detection; LLOQ, lower limit of quantification
