# Erratum to: Long-term monitoring of opioid, sedative and anti-inflammatory drugs in horse hair using a selective and sensitive LC-MS/MS procedure

**DOI:** 10.1186/s12917-016-0746-0

**Published:** 2016-06-22

**Authors:** Milena M. Madry, Barbara S. Spycher, Jacqueline Kupper, Anton Fuerst, Markus R. Baumgartner, Thomas Kraemer, Hanspeter Naegeli

**Affiliations:** Zurich Institute of Forensic Medicine, Center for Forensic Hair Analytics, University of Zurich, Zurich, Switzerland; Zurich Institute of Forensic Medicine, Center for Forensic Pharmacology and Toxicology, University of Zurich, Zurich, Switzerland; Institute of Veterinary Pharmacology and Toxicology, University of Zurich, Zurich, Switzerland; Clinic of Veterinary Surgery, Department of Large Animal Surgery, University of Zurich, Zurich, Switzerland

## Erratum

Unfortunately, after publication of this article [1], it was noticed that the axis numbering of Fig. 4 was switched. The numbering of the x-axis should be on the y-axis and vice versa. The corrected figure can be seen below.

**Fig. 4:**
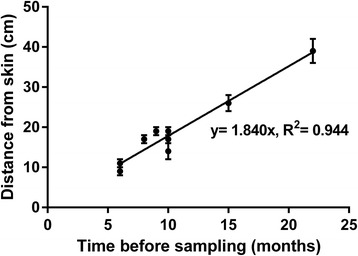
Drug localization in horse hair as a function of the time of documented drug administrations. This graph shows the median distance from skin and range of segments with clearly increased drug incorporation. The slop of the resulting linear relationship is consistent with an average growth rate of 1.84 cm per month
